# Intraspecies Variation in the Emergence of Hyperinfectious Bacterial Strains in Nature

**DOI:** 10.1371/journal.ppat.1002647

**Published:** 2012-04-12

**Authors:** Douglas M. Heithoff, William R. Shimp, John K. House, Yi Xie, Bart C. Weimer, Robert L. Sinsheimer, Michael J. Mahan

**Affiliations:** 1 Department of Molecular, Cellular and Developmental Biology, University of California, Santa Barbara, California, United States of America; 2 University of Sydney, Faculty of Veterinary Science, Camden, New South Wales, Australia; 3 Department of Population Health and Reproduction, School of Veterinary Medicine, University of California, Davis, California, United States of America; University of Washington, United States of America

## Abstract

*Salmonella* is a principal health concern because of its endemic prevalence in food and water supplies, the rise in incidence of multi-drug resistant strains, and the emergence of new strains associated with increased disease severity. Insights into pathogen emergence have come from animal-passage studies wherein virulence is often increased during infection. However, these studies did not address the prospect that a select subset of strains undergo a pronounced increase in virulence during the infective process- a prospect that has significant implications for human and animal health. Our findings indicate that the capacity to become hypervirulent (100-fold decreased LD_50_) was much more evident in certain *S. enterica* strains than others. Hyperinfectious salmonellae were among the most virulent of this species; restricted to certain serotypes; and more capable of killing vaccinated animals. Such strains exhibited rapid (and rapidly reversible) switching to a less-virulent state accompanied by more competitive growth ex vivo that may contribute to maintenance in nature. The hypervirulent phenotype was associated with increased microbial pathogenicity (colonization; cytotoxin production; cytocidal activity), coupled with an altered innate immune cytokine response within infected cells (IFN-β; IL-1β; IL-6; IL-10). Gene expression analysis revealed that hyperinfectious strains display altered transcription of genes within the PhoP/PhoQ, PhoR/PhoB and ArgR regulons, conferring changes in the expression of classical virulence functions (e.g., SPI-1; SPI-2 effectors) and those involved in cellular physiology/metabolism (nutrient/acid stress). As hyperinfectious strains pose a potential risk to human and animal health, efforts toward mitigation of these potential food-borne contaminants may avert negative public health impacts and industry-associated losses.

## Author Summary

Salmonellosis continues to compromise human health, animal welfare, and modern agriculture. Developing a comprehensive control plan requires an understanding of how pathogens emerge and express traits that confer increased incidence and severity of disease. It is well-established that animal passage often results in increased virulence; however, our findings indicate that the capacity to undergo a pronounced increase in virulence after passage was much more prevalent in certain *Salmonella* isolates than in others. The resultant hyperinfectious strains are among the most virulent salmonellae reported; were restricted to certain serotypes; and were able to override the immunity conferred in vaccinated animals. The induction of hypervirulence was responsive to subtle changes in environmental conditions and, potentially, may occur in other salmonellae serotypes after passage through certain hosts and/or exposure to certain environmental variables; a response that may be common across the microbial realm. Thus, management practices and environmental conditions inherent to livestock production have the potential to inadvertently trigger hypervirulence (e.g., diet; herd size; exposure to livestock waste and/or antimicrobials). From a farm management perspective, careful consideration must be given to risk-management strategies that reduce emergence/persistence of these potential food-borne contaminants to safeguard public health and reduce industry-associated losses.

## Introduction

*Salmonella enterica* is a significant food-borne pathogen of humans causing up to an estimated 1.3 billion cases of disease worldwide, annually [1], [2]. *S. enterica* is acquired via the fecal-oral route and is comprised of six subspecies that are subdivided into more than 2500 serovars (serological variants) based on carbohydrate, lipopolysaccharide (LPS), and flagellar composition [2]. *S. enterica* infection can result in any of four distinct syndromes: enterocolitis/diarrhea, bacteremia, enteric (typhoid) fever, and chronic asymptomatic carriage [2]–[4]. Many serovars infect both humans and animals wherein the particular syndrome and disease severity is a function of the serovar and host susceptibility [5], [6].

Such host-susceptibility differences present a formidable challenge to the design of salmonellae control strategies for a number of reasons: 1) Most infections of livestock are subclinical as evidenced by the disparity between the frequency and diversity of isolates from surveillance and clinical submissions [7]–[9]; 2) Some isolates are capable of asymptomatic colonization and/or persistence in a particular animal species while causing acute disease in another animal species (e.g., different types or classes of stock) [2]–[4]; 3) Although a diversity of serotypes are frequently isolated from intensive livestock production systems, disease outbreaks are often intermittent and associated with specific serotypes [8]–[10]; 4) The capacity of salmonellae to survive and proliferate in the environment provides a large dynamic reservoir for infection of livestock and a vehicle for cross-contamination from animal to human food products [11]–[14]. These factors are of particular relevance to the global trend toward intensive livestock production that favors fecal-oral pathogen transmission, and the resultant increased risk of animal disease and contamination of livestock-derived food products [8]–[10], [15].

The diversity of salmonellae present on farms and feedlots, and the potential for different serovars to possess an array of virulence attributes, necessitates the use of broad prophylactic strategies that are efficacious for many serovars simultaneously. An effective approach for a number of years has been the therapeutic and prophylactic administration of antibiotics to livestock, but this option has become limited due to the emergence of multi-drug resistant pathogenic strains that also present a bona fide risk to human health [1], [9], [16]. Vaccination is one of the best forms of prophylaxis against the development of disease caused by infectious agents. Although vaccination is generally highly specific in the protection conferred in immunized hosts (protection is limited to a specific strain or closely-related set of strains), recent advancements have resulted in the development of vaccines that elicit cross-protective immunity to multiple strains of the same species [17]–[21]. However, currently available vaccines may elicit limited protection against new pathogens that may express traits that confer enhanced virulence or compromised host immunity.

The continuing emergence of new virulent strains associated with an increased incidence and/or severity of disease has yet to be explained. Insights have been derived from prior animal-passage studies wherein virulence traits often are increased (reversibly) following animal passage (e.g., accelerated colonization; hastened morbidity/mortality; reviewed in [22]–[24]). For example, host passage of *Vibrio cholerae* [25] and *Citrobacter rodentium* [26] results in the transition to a hypervirulent state that is maintained for a limited time after fecal shedding and may contribute to epidemic spread of the organism [27]. Further, epidemiological evidence indicates that animals can be infected by natural transmission (via direct contact with infected animals) with a significantly lower infectious dose than with organisms obtained from laboratory culture (e.g., *E. coli* O157:H7 and *S.* Choleraesuis) [28]–[30]. However, many animal passage studies were performed on a limited number of strains; often only a modest increase in virulence was observed; multiple rounds of animal passage were required; and did not address the prospect that animal passage may lead to markedly increased virulence in some strains and hosts but not others [25], [26], [31]–[39].

In this study, a collection of *Salmonella* clinical isolates was screened for those that, following infection, exhibited a pronounced increase in virulence relative to other passaged isolates. Some salmonellae strains exhibited the hypervirulent phenotype after in vivo passage, whereas others did not, indicating intraspecies variation in the capacity for their development. The resultant hyperinfectious strains were among the most virulent salmonellae reported and were subsequently shown to be more capable of infecting vaccinated animals.

## Materials and Methods

### Strains and media

*Salmonella* human clinical isolates were obtained from fecal and blood samples derived from patients with gastroenteritis or bacteremia, respectively; animal isolates were derived from different outbreaks, individual cases, or surveillance submissions to diagnostic laboratories [20]. Virulent *S.* Typhimurium reference strain ATCC 14028 (CDC 6516-60) was used in all studies for comparison. Unless otherwise specified, bacteria were derived from stationary phase cultures aerated at 37°C containing either Luria-Bertani (LB) medium [40] or low phosphate, low magnesium, pH 5.5 medium supplemented with 0.3% glycerol and 0.1% casamino acids (LPM pH 5.5) [41], [42].

### Ethics statement

All animal experimentation was conducted following the National Institutes of Health guidelines for housing and care of laboratory animals and performed in accordance with Institutional regulations after pertinent review and approval by the Institutional Animal Care and Use Committee at the University of California, Santa Barbara.

### Virulence assays

*Oral and Intraperitoneal Lethal Dose_50_ (LD_50_):* The dose required to kill 50% of infected animals was determined via the oral (via gastrointubation) and intraperitoneal (i.p.) routes by infecting at least 10 mice [43]. *Salmonella* test strains and wild-type *S.* Typhimurium reference strain 14028 were grown overnight in LB or LPM pH 5.5 medium. Bacterial cells resuspended in 0.2 ml of 0.2M Na_2_HPO_4_ pH 8.1 or 0.1 ml of 0.15M NaCl (for oral and i.p. administration, respectively) were used to infect mice, which were examined daily for morbidity and mortality up to 3 weeks post-infection. The oral and i.p. LD_50_ for wild-type *S.* Typhimurium reference strain 14028 is 10^5^ and <10 organisms, respectively [43]. *Competitive Index (CI):* The CI value is the relative in vivo recovery ratio of test strain/reference strain obtained from target tissues after equivalent doses are co-administered by i.p. infection [44]. *Salmonella* test strains were grown overnight in either LB or LPM pH 5.5 medium; *S.* Typhimurium reference strain MT2057 (a virulent derivative of strain 14028) was grown in LB [43], [44]. Bacterial cells were resuspended in 0.15M NaCl and an equivalent dose (500 bacterial cells) of a test strain and *S.* Typhimurium reference strain MT2057 was co-administered i.p. to at least 5 mice. Five days post-infection, the bacterial cells were recovered from the spleen of acutely infected animals. The CI value is the ratio of test strain/reference strain recovered from the spleen divided by the ratio of the input inoculum; bacterial cell number was enumerated by direct colony count. *S.* Typhimurium reference strain MT2057 (used in the CI studies) is a virulent derivative of strain 14028, containing a Lac^+^ Mu*d*J transcriptional fusion which is used to discern it from other *Salmonella* that are inherently Lac^−^. Note that the oral and i.p. LD_50_ (10^5^ and <10 organisms, respectively), as well as the i.p. competitive index, of strain MT2057 are indistinguishable from that of the parental wild-type strain, 14028 [43], [44]. Six- to- eight week old BALB/c mice were used in all virulence studies.

### Screen for hyperinfectious strains

A collection of 184 *Salmonella* human and animal clinical isolates [20] cultured in rich medium was screened for those that were initially attenuated for virulence via the i.p. route of infection (10^3^- fold decreased i.p. CI; 10- fold increased i.p. LD_50_); that harbored the virulence plasmid necessary for systemic disease [45], [46]; and that were competent for virulence via the oral route of infection (oral LD_50_ of 10^5^ cells). The 14 isolates that answered this screen were subjected to oral animal passage whereby bacteria (10^9^ cells) derived from stationary phase cultures containing LB medium were used to perorally infect mice. Five to seven days post-infection, spleens were aseptically removed from acutely infected mice, homogenized in 1 ml of 0.2M Na_2_HPO_4_ pH 8.1 (10^8^ to 10^9^ CFU/g of spleen), and used, without ex vivo growth, to infect naïve animals at doses equivalent to, and 10- to 100- fold lower than, the oral LD_50_ of the same strain grown in LB medium (10^5^ cells). Such animal passage resulted in the development of hyperinfectious strains for all (14/14) isolates tested, as confirmed by a 10- to 100- fold reduced oral and i.p. LD_50_ and a 10^3^- to 10^4^- fold increased i.p. CI relative to the values attained after growth in LB medium. Mice were examined daily following infection for morbidity and mortality up to 3 weeks post-infection.

### Cell culture

The murine macrophage cell line RAW 264.7 (ATCC TIB-71) was obtained from the American Type Culture Collection, Rockville, MD., and maintained in minimum essential medium (MEM) supplemented with L-glutamine and 10% heat-inactivated bovine growth-supplemented calf serum (HyClone Laboratories, Logan, UT). Cells were grown in a humidified atmosphere of 5% carbon dioxide and 95% air at 37°C in 75-cm^2^ plastic flasks (Corning Glass Works, Corning, NY). Cultured murine macrophages (RAW 264.7) were harvested by scraping with a rubber policeman and plated at a density of 2.5×10^5^ to 5×10^5^ cells/ml in 4 ml of culture medium in 35 mm-diameter, six-well dishes (Corning) and grown for 24 h to approximately 80 to 90% confluence (1×10^6^ to 5×10^6^ cells/well) (adapted from [47]).

### Bacterial infection of cultured murine macrophages

Bacterial cells were used to infect cultured murine macrophage (RAW 264.7) monolayers grown in cell culture plates (Corning) at a multiplicity of infection (MOI) of 10∶1 or 100∶1. The bacteria were centrifuged onto cultured monolayers at 1,000× *g* for 10 min at room temperature, after which they were incubated for 30 min at 37°C in a 5% CO_2_ incubator (t = 0 time point). The coculture was washed once with cell culture medium and incubated for 45 min in the presence of gentamicin (100 µg/ml) to kill extracellular bacteria, washed once with pre-warmed cell culture medium, and incubated with gentamicin (10 µg/ml) to the time points indicated (adapted from [48]).

### Bacterial cytocidal activity assay

Macrophage (RAW264.7) cell viability following *Salmonella* infection was quantified via a crystal violet dye retention assay in 96 well-plates adapted from references [49], [50]. Bacteria derived from stationary phase cultures containing either LB or LPM pH 5.5 medium were used to infect cultured macrophage monolayers (5×10^4^ to 1×10^5^ cells/well) at an MOI of 10∶1 or 100∶1 as described above. At 20 h post-infection, the monolayer cultures were rinsed twice with PBS, and the adherent cells were fixed and stained for 10 min with 0.2% crystal violet in 20% methanol. Monolayers were washed three times with phosphate buffered saline (PBS) to remove excess crystal violet. Dye retained by the cells was released using a 50% ethanol/0.1% acetic acid mixture, diluted 1∶2 in PBS, and quantified by absorbance at 577 nm. High cytocidal activity is associated with low dye retention and vice versa. Data given are representative absorbance values derived from each condition performed in triplicate. Standard error of triplicate means is <20%.

### Quantitation of macrophage cytokines post-infection via qPCR analysis

Bacteria grown overnight in LB or in LPM pH 5.5 medium were used to infect cultured macrophage (RAW264.7) monolayers at an MOI of 10∶1 in 6-well culture plates as described above. Total RNA was prepared using the RNeasy Mini kit (Qiagen) as specified by the manufacturer's protocol. RNA concentrations were determined spectrophotometrically. Reverse transcription was carried out using 2 µg of total RNA with the Superscript cDNA Synthesis Kit (Invitrogen) as per the manufacturer's protocol. qPCR was performed using iQ SYBR Green Supermix (BioRAD) and an iQ5 real time PCR thermocycler (BioRAD). For amplification of mouse genes, the primer pairs were those described in the following studies: IFN-β [51]; IL-1β, IL-6 and IL-10 [52]; iNOS and GAPDH [53]. Quantification of the qPCR product was carried out using the iQ5 optical system software (BioRAD). All target gene transcripts were normalized to that of the GAPDH gene. The expression ratio value is the level of transcripts obtained from infected relative to uninfected cells.

### Transcriptome analysis

#### Bacterial RNA/cDNA preparation

Bacterial strains were grown overnight with aeration at 37°C in LB broth, pelleted and washed in 0.15M NaCl, and split without dilution into two cultures containing either LB or LPM pH 5.5 medium. The cultures were incubated with aeration at 37°C for 4 h after which approximately 2.5×10^10^ cells were pelleted via centrifugation, snap-frozen in an ethanol-dry ice bath, and stored at −80°C. Bacterial cell pellets were lysed using Max Bacterial Enhancement Reagent (Invitrogen) at 95°C for 5 min. Total bacterial RNA (≥10 µg) was isolated using TRIzol Max Bacterial Isolation Kit (Invitrogen), and purified with an RNAeasy MinElute kit with on-column DNase digestion (Qiagen) (A_260/280_ ratio of ≥2.0 and an A_260/230_ ratio of ≥1.5). Reverse transcription of total RNA was carried out using Superscriptase II and random hexamers (Invitrogen). After NaOH treatment to eliminate the RNA template, single-stranded cDNA was purified with QIAquick PCR MinElute purification kit (Qiagen).

#### Array design and hybridization

cDNA (1 µg) was sheared for 10 min with 0.6 U of DNase I at 37°C (Promega, WI); and labeled with a custom GeneChip DNA designed by B. C. Weimer (UC Davis) in conjunction with Affymetrix Inc. (Santa Clara, CA). Genomic DNA (50 ng) was labeled according to the *Escherichia coli* protocol and hybridized onto custom Affymetrix DNA chips containing probe sets designed for all the annotated coding sequences (CDSs) and intergenic spaces of *S.* Typhimurium LT2 genome, resulting in 4,510 probe sets composed of 11 unique 25-mer probe sequences per CDS. The chips were hybridized and scanned at the Center for Integrated BioSystems (Utah State University, Logan, UT), according to the manufacturer's protocols for *E. coli*. Hybridizations for each strain were performed in two biological replicates.

#### Data normalization, visualization, and analysis

Gene expression analysis was performed to identify bacterial gene transcripts that were significantly altered in hyperinfectious strains under LB versus LPM pH 5.5 conditions, and not altered, or altered to the same extent, in a conventionally virulent strain. Raw probe-level intensities (.cel files) from all chips were background corrected using the robust multichip average (RMA) method, normalized using loess, and summarized using the Bioconductor Affy package. The raw log_2_ gene-level Affymetrix expression values were transformed to produce log_2_ LPM/LB ratio values for conventionally virulent *S.* Typhimurium (ST), and hypervirulent *S.* Bovismorbificans (SB) and *S.* Choleraesuis (SC) strains. Subsequently, log_2_ LPM/LB ratio data were loaded into the CLC Genomics Workbench and further normalized (CLC bio, Cambridge, MA); and the log_2_ LPM/LB ratio statistical differences between conventionally and hypervirulent strains were evaluated using the CLC Expression analysis module with SB and SC grouped together. Two criteria were used as a cutoff to identify the genes that were significantly altered in hyperinfectious strains under LB versus LPM pH.5.5 conditions, and not altered, or altered to the same extent, in a conventionally virulent strain; i.e., at least a 2-fold expression change in SB, SC or ST; and a 0.05 false discovery rate (FDR) when comparing log_2_ LPM/LB ratios values for SB and SC versus ST. Heat maps were generated from the resultant list of genes using The Institute for Genomic Research MultiExperiment Viewer (MeV), version 4.7 [54]. Unsupervised data analysis was performed in MeV using hierarchical clustering (HCL) [55] modules. All expression experiments were done in two biological replications.

### Statistical analyses

#### Mouse disease susceptibility

The disease susceptibility in vaccinated mice infected with hyperinfectious and conventionally virulent salmonellae was determined by comparing the proportion of mice surviving virulent challenge using Chi-square (Epicalc 2000 version 1.02, 1998 Brixton Books).

#### Bacterial cytocidal activity

Cytocidal activity of hyperinfectious and conventionally virulent salmonellae upon infection of cultured macrophages was subjected to analysis of variance in GenStat (13^th^ edition, VSN International Ltd, Hemel Hempstead, UK) using a model that had serotype, media, and dose as the main effects. The change in cytocidal activity of hyperinfectious strains (*S.* Choleraesuis χ3246 and *S.* Bovismorbificans 158) was individually contrasted to the change in cytocidal activity of reference *S.* Typhimurium strain 14028 at each dose level according to the following ‘a priori’ contrast: cytocidal activity of the hyperinfectious serovar grown in LB medium minus the cytocidal activity grown in LPM medium versus the cytocidal activity of *S.* Typhimurium 14028 grown in LB medium minus the cytocidal activity of *S.* Typhimurium 14028 grown in LPM.

#### Innate immune cytokine response

Differences in gene expression displayed by infected relative to uninfected murine macrophage values were analyzed using residual (or restricted) maximum likelihood (REML) analysis (Genstat, 13^th^ Edition, VSN International Ltd, Hemel Hempstead, UK). A single variate, repeated measures model was fitted for the factors media, organism and time. The Wald chi-square test was used to determine significant individual effects and interactions between factors. Differences between the individual means were determined by calculating an approximate least significant difference (LSD), using predicted model-based means. Predicted means are those obtained from the fitted model rather than the raw sample means, as predicted means represent means adjusted to a common set of variables, thus allowing valid comparison between means. A difference of means that exceeded the calculated LSD was considered significant. For all statistical analyses, a significance level (*P*) of less than 0.05 was considered to be statistically significant.

#### Gene expression analysis

A description of the transcriptome statistical analysis is provided in the previous *Materials and Methods* section under data normalization, visualization, and analysis.

## Results

### Screen for *Salmonella* strains that exhibit a pronounced increase in virulence following infection relative to other animal-passaged isolates

A collection of 184 *Salmonella* clinical isolates was obtained from fecal and blood samples derived from human patients with gastroenteritis or bacteremia; and from animal isolates derived from different outbreaks, individual cases, or surveillance submissions to diagnostic laboratories [20]. These isolates were cultured in rich (LB) medium and screened for those that *i)* were attenuated for virulence via the i.p. route of infection (10^3^-fold decreased i.p. CI; 10-fold increased i.p. LD_50_); *ii)* harbored the virulence plasmid necessary for systemic disease [45], [46]; and *iii)* were competent for virulence via the oral route of infection (oral LD_50_ of 10^5^ cells). The fourteen isolates that answered this screen were grown overnight in LB medium and used to perorally infect mice. Five to seven days post-infection, bacteria derived from spleens harvested from the resultant acutely infected animals were used, without ex vivo growth, to orally infect naïve animals at doses equivalent to, and 10- to 100-fold lower than, the oral LD_50_ of the same strain grown in LB medium (10^5^ cells). The prior in vivo passage resulted in the development of hyperinfectious strains for all (14/14) isolates tested, as evidenced by a 10- to 100- fold reduced oral and i.p. LD_50_ and a 10^3^- to 10^4^- fold increased i.p. CI relative to the values attained after growth in LB medium (Table 1). These isolates comprise some of the most virulent salmonellae strains reported (i.e., oral LD_50_ of 10^3^ organisms). In contrast, although in vivo passage of other clinical isolates exhibited increased virulence traits after murine passage (increased colonization; decreased time to morbidity/mortality)- a phenomenon shown previously [39] and recapitulated here, none (0/7) exhibited a marked change in LD_50_ or CI value relative to that attained after in vitro growth. This was also the case for conventionally virulent *Salmonella* reference strain 14028. Taken together, these data indicate that the 14 hyperinfectious *Salmonella* strains are considerably more virulent than other animal-passaged clinical isolates (100-fold decreased LD_50_); and the display of increased virulence traits by bacterial strains after murine passage does not necessarily equate to hypervirulence.

**Table 1:** Comparison of virulence states between hyperinfectious salmonellae and other clinical isolates following laboratory culture and animal passage.

		In vitro passage^b^			In vivo passage^b^		
Strain^a^	Serovar	Oral LD_50_ c	i.p. LD_50_ c	Competitive index^d^	Oral LD_50_	i.p. LD_50_	Competitive index
*Hyperinfectious strains*							
χ3246	*S.* Choleraesuis	10^5^	10^2^	3.0×10^−4^	10^3^	<10^1^	6.2
3	*S.* Choleraesuis	10^5^	10^2^	<3.0×10^−4^	10^4^	<10^1^	0.6
(03)-6339	*S.* Choleraesuis	10^5^	10^2^	<3.0×10^−4^	10^3^	<10^1^	2.4
58	*S.* Bovismorbificans	10^5^	10^2^	<3.0×10^−4^	10^4^	<10^1^	3.0
158	*S.* Bovismorbificans	10^5^	10^2^	<3.0×10^−4^	10^3^	<10^1^	1.5
208	*S.* Bovismorbificans	10^5^	10^2^	<3.0×10^−4^	10^3^	<10^1^	1.8
*Other clinical isolates*							
Lane	*S.* Dublin	10^5^	<10^1^	0.6	10^5^	<10^1^	0.4
4973	*S.* Enteritidis	10^5^	<10^1^	1.3	10^5^	<10^1^	9.0
F98	*S.* Typhimurium	10^5^	<10^1^	0.5	10^5^	<10^1^	0.8
UK-1	*S.* Typhimurium	10^5^	<10^1^	2.4	10^5^	<10^1^	0.7
14028	*S.* Typhimurium ref. strain	10^5^	<10^1^	0.8	10^5^	<10^1^	4.6

a All (184) *Salmonella* human and animal isolates tested were recovered from different outbreaks or individual cases submitted to diagnostic laboratories, or from surveillance submissions of on-farm healthy animals [20]. Eighty-one of these strains harbored the virulence plasmid necessary for systemic disease [46] but exhibited an i.p. virulence defect in a mouse model of typhoid fever; of these isolates, 14 were virulent by the oral route of infection. Conventionally virulent *S.* Typhimurium reference strain 14028 was used in all studies for comparison.

b In vitro/in vivo passage. *In vitro passage*. Bacteria derived from overnight stationary phase cultures containing LB medium were used to infect BALB/c mice via the oral or i.p. route of infection as described in *Materials and Methods*. *In vivo passage*. Bacteria (10^9^ cells) derived from stationary phase cultures containing LB medium were used to orally infect mice. Five to seven days post-infection, bacteria derived from spleens harvested from acutely infected animals (10^8^ to 10^9^ CFU/g of spleen determined by direct colony count) were used, without ex vivo growth, to infect naïve mice via the oral or i.p. route of infection as described in *Materials and Methods*.

c LD_50_ virulence assay. The dose required to kill 50% of infected animals (LD_50_) was determined via the oral (via gastrointubation) and i.p. routes by infecting at least 10 mice as described in *Materials and Methods*.

d Competitive Index (CI) virulence assay. An equivalent dose (500 bacterial cells) of a test strain and a Lac^+^ derivative of *S.* Typhimurium reference strain 14028 (MT2057) was co-administered i.p. to at least 5 mice; the CI value is the ratio of test strain/reference wild-type strain recovered from target tissue (spleen) divided by the input ratio [28] as described in *Materials and Methods*.

### Intraspecies variation in the development of hyperinfectious salmonellae strains

Most cases of human and livestock salmonellosis are caused by one *Salmonella* subspecies, termed *S. enterica* subsp. *enterica* [9], [56]–[59]. Here we examined whether there was variation within subsp. *enterica* serovars in the capacity for the development of hyperinfectious strains following murine passage. Our data show that the hypervirulent phenotype was much more evident in some subsp. *enterica* serovars (*S.* Bovismorbificans [11/11]; *S.* Choleraesuis [3/3]) (serogroups C2-C3 and C1, respectively), than others (*S.* Typhimurium [0/52]; *S.* Dublin [0/8]; *S.* Enteritidis [0/7]) (serogroups B, D, and D, respectively) (*P*<0.01). These data suggest that, following murine infection, *Salmonella* serovars exhibit intraspecies variation in the development of hyperinfectious strains.

### Hyperinfectious salmonellae exhibit distinct colonization kinetics relative to that of other animal passaged isolates

**Figure 1:**
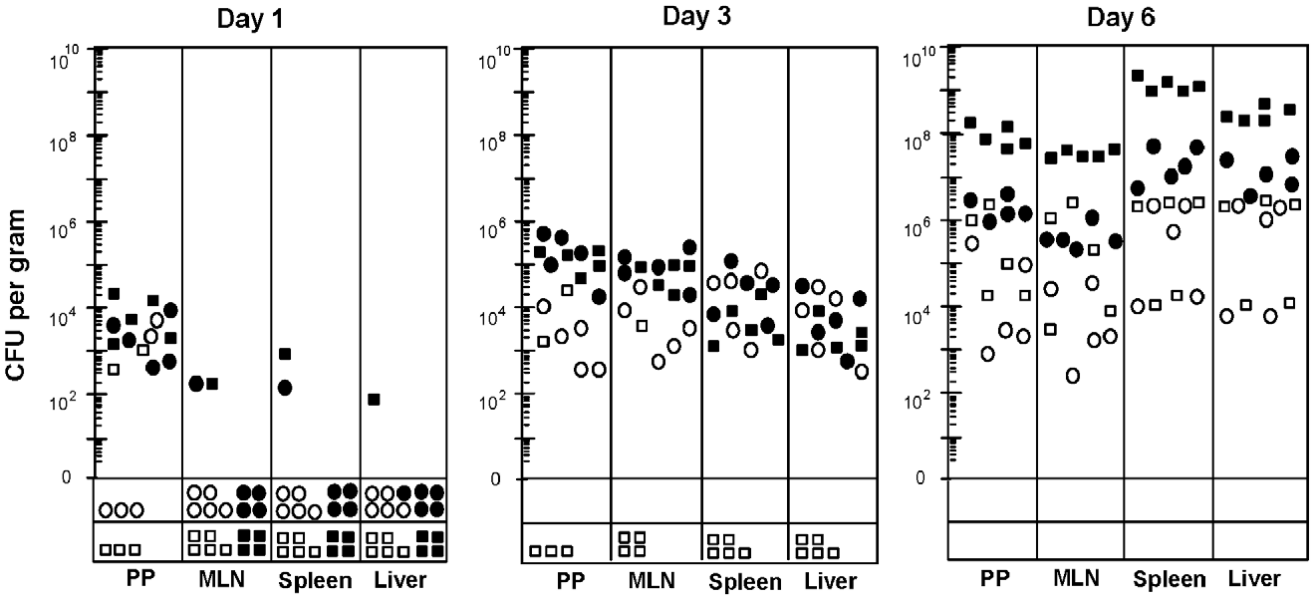
Comparison of host site colonization between hyperinfectious and conventionally virulent salmonellae following laboratory culture and animal passage. BALB/c mice were infected orally (10^7^ CFU) with hyperinfectious *S.* Choleraesuis χ3246 (boxes) or conventionally virulent *S.* Typhimurium reference strain 14028 (circles). These bacterial cells were derived from either stationary phase cultures containing LB medium (open symbols); or after in vivo passage (closed symbols), whereby 5 to 7 days post- oral infection, spleens were aseptically removed from acutely infected mice, and used, without ex vivo growth, to orally infect naïve animals. PP, Peyer's Patches; MLN, mesenteric lymph nodes; CFU, colony forming units. The symbols below the zero CFU value represent the number of mice in which the bacterial load was below the limits of detection: PP, MLN, spleen <40 CFU; Liver <20 CFU.

To determine the spatio-temporal nature of the development of hyperinfectious strains, the kinetics of host tissue colonization was followed throughout the infective process. Upon oral infection, hyperinfectious *S.* Choleraesuis χ3246 grown in LB medium exhibited a pronounced lag in colonization of mucosal tissues and visceral organs and did not attain the high bacterial load exhibited by the same strain after murine passage (open versus closed boxes; Figure 1). In contrast, conventionally virulent *Salmonella* reference strain 14028 grown in LB medium did not display the pronounced lag in colonization exhibited by *S.* Choleraesuis χ3246 (open circles versus open boxes). Further, although murine-passaged *S.* Typhimurium 14028 exhibited increased colonization (open versus closed circles) as has been observed with *Salmonella* and other enteric pathogens [25], [26], [39], [60], its passage did not result in the high bacterial load exhibited by murine-passaged *S.* Choleraesuis χ3246 at late stages of infection (closed symbols), nor was it associated with the pronounced decrease in LD_50_ associated with hyperinfectious strains after passage (Table 1). These data indicate that hyperinfectious strains undergo a switch from a less-virulent to hypervirulent state following a pronounced lag during the infective process, and the resultant hyperinfectious strains are much more virulent than other animal-passaged clinical isolates.

### Hyperinfectious salmonellae can be isolated under defined conditions in vitro, and adopt distinct virulence states depending on prior growth conditions

Next, we questioned whether strains that exhibited the hypervirulent phenotype in vivo also had the capacity to enter the hypervirulent state under defined conditions in vitro. Efforts were initially focused on conditions reported to reflect that of the macrophage phagosome, a principal organelle in which salmonellae reside during infection [61], [62]; such conditions are characterized by low phosphate, low magnesium and mildly acidic medium (LPM pH 5.5) [41], [42]. Growth of *S.* Choleraesuis χ3246 and *S.* Bovismorbificans 158 in LPM pH 5.5 medium resulted in the recovery of hyperinfectious strains similar to those obtained after murine passage, as evidenced by a 100- fold reduced oral LD_50_ and a 10^4^- fold increased i.p. CI value relative to that obtained after growth in LB medium (Table 2). Further, the degree of virulence exhibited by the hyperinfectious strains was exquisitely sensitive to prior growth conditions resulting in low-, medium-, and high- virulence states as evidenced by the varied i.p. CI values exhibited in the four media tested (LB; LPM pH 5.5; LPM pH 7.0; minimal medium pH 5.5). In contrast, growth of conventionally virulent *S.* Typhimurium reference strain 14028 in LPM pH 5.5 conditions did not result in a pronounced increase in virulence relative to LB medium, nor was the degree of virulence markedly dependent on prior growth conditions as evidenced by similar i.p. CI values in the four media tested. These data indicate that the hypervirulent phenotype can be fully recapitulated in vitro, and hyperinfectious strains are capable of adopting widely disparate virulence states depending on growth conditions. Such variability was not observed with conventionally virulent *S.* Typhimurium 14028.

**Table 2:** Comparison of virulence states between hyperinfectious and conventionally virulent salmonellae following growth under defined laboratory conditions.

		Oral LD_50_		i.p. CI			
Strain^a^	Serovar	LB	LPM pH 5.5	LB	LPM pH 5.5	LPM pH 7.0	Minimal pH 5.5
χ3246	*S.* Choleraesuis	10^5^	10^3^	3.0×10^−4^	5.0	9.6×10^−4^	5.2×10^−2^
158	*S.* Bovismorbificans	10^5^	10^3^	<3.0×10^−4^	1.3	1.3×10^−1^	8.6×10^−2^
14028	*S.* Typhimurium ref. strain	10^5^	10^5^	0.8	2.5	0.8	3.6

a BALB/c mice were orally or i.p. infected with hyperinfectious *S.* Choleraesuis χ3246, *S.* Bovismorbificans 158 or conventionally virulent *S.* Typhimurium reference strain 14028 derived from stationary phase cultures containing either LB; low phosphate low magnesium (LPM pH 5.5) [41], [42]; or minimal E medium [40] supplemented with 0.2% glucose and 0.1% casamino acids, at the pH indicated. Oral LD_50_ and i.p. competitive index (CI) virulence assays were performed as in Table 1.

### The induction of hypervirulence is rapid and rapidly reversible, and does not require vigorous bacterial cell growth

**Figure 2:**
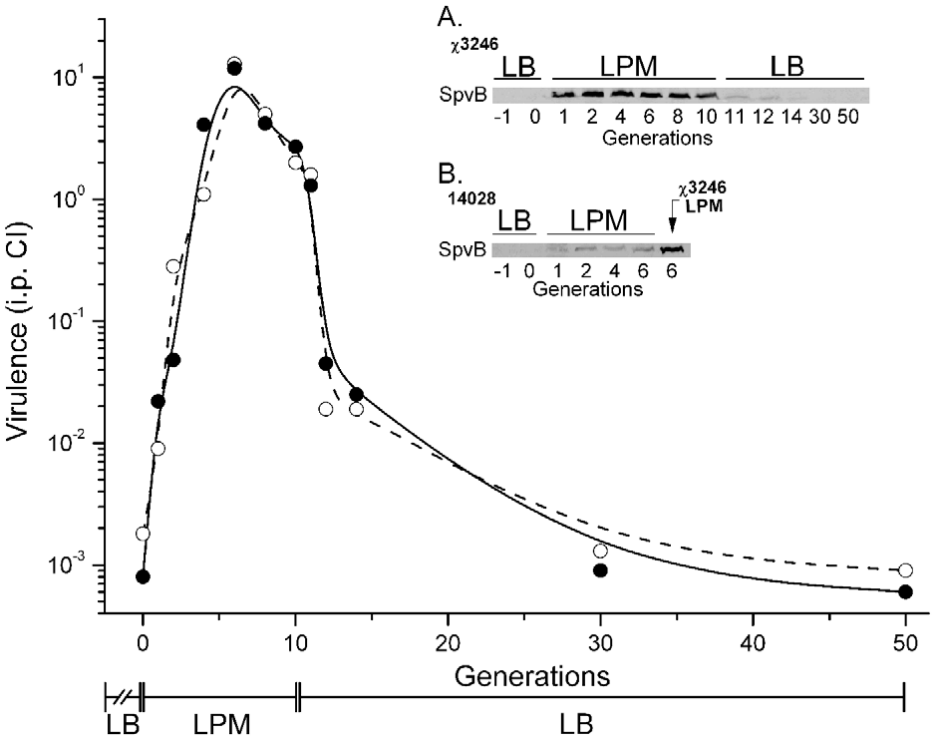
Comparison of the degree of virulence and cytotoxin production in hyperinfectious salmonellae following media shift to permissive conditions for hypervirulence. The degree of virulence and *Salmonella* SpvB cytotoxin [63], [64] production were assessed as a function of growth under conditions that were permissive (LPM pH 5.5 medium) or non-permissive (LB medium) for the hypervirulent phenotype. Insert A. Hyperinfectious *S.* Choleraesuis χ3246 grown in LB medium was transferred to LPM pH 5.5 medium for 10 cell generations (cell doublings); subsequently, such cells were transferred back into LB medium for 40 cell generations. Bacterial cells were obtained from, and maintained in, exponential phase cultures diluted periodically such that the cell number was constant at each sampling point. Cell aliquots at the time points indicated were assessed for virulence via i.p. competitive index (CI) virulence assays in two independent experiments (open and closed circles) and for SpvB cytotoxin production (representative sample). SpvB cytotoxin was evaluated via whole cell protein extracts corresponding to ∼7×10^7^ *Salmonella* cells subjected to SDS-PAGE, and transferred to PVDF membrane. Insert B. Conventionally virulent *S.* Typhimurium reference strain 14028 and hyperinfectious *S.* Choleraesuis χ3246 grown in LB medium were transferred to LPM pH 5.5 medium for 6 cell generations; SpvB cytotoxin was evaluated via whole cell protein extracts corresponding to ∼2×10^7^ *Salmonella* cells subjected to SDS-PAGE, and transferred to PVDF membrane. Membranes were probed with *Salmonella* rabbit anti-SpvB (Don Guiney, UCSD), and an infrared (IR) dye-conjugated donkey anti-rabbit immunoglobulin G (IRDye 800CW, Li-Cor Biosciences) was used as secondary antibody. Signal was detected using an Odyssey IR imaging system (Li-Cor Biosciences).

Targeting of the actin cytoskeleton during infection by the *Salmonella* SpvB cytotoxin promotes intracellular survival, host cell cytotoxicity, and bacterial dissemination [63], [64]. To understand the mechanistic nature of switching between less-virulent and hypervirulent states, the kinetics of hypervirulence and *Salmonella* cytotoxin (SpvB) production were assessed upon transfer from nonpermissive (LB medium) to permissive (LPM pH 5.5 medium) conditions for the hypervirulent phenotype. Transfer of *S.* Choleraesuis χ3246 from LB to LPM pH 5.5 medium resulted in a rapid transformation from the virulence-attenuated to the hypervirulent phenotype, as evidenced by a 10^4^-fold increase in i.p. CI value 6- to 8- cell generations (cell doublings) post-transfer (Figure 2). This was accompanied by a 50-fold increase in SpvB production within 1- to 2- cell generations post-transfer (Figure 2; inset A). SpvB production was also stimulated in *S.* Typhimurium 14028 upon transfer from LB to LPM pH 5.5 medium, as was shown previously after bacterial entry into macrophages and epithelial cells [65]. However, the resultant protein levels were 8-fold less than that of *S.* Choleraesuis χ3246 (Figure 2; inset B). Further, since SpvB production in *S.* Choleraesuis χ3246 occurred more rapidly than that observed for virulence upon media shift, the full impact of cytotoxin levels on virulence is either delayed and/or other virulence factors are necessary for the hypervirulent phenotype. Upon subsequent transfer from LPM pH 5.5 medium back to LB medium, the hypervirulent phenotype and associated cytotoxin production was rapidly reversible to a less-virulent state, as evidenced by a 500-fold decrease in i.p. CI value and a 30-fold reduction in SpvB within four generations, and a further return to levels exhibited by parental cells after 20- to 40- cell generations. The rapid and rapidly reversible nature of the hypervirulent phenotype suggests that a non-mutational mechanism controls the switching between less-virulent and hypervirulent states.

We then examined whether induction of the hypervirulent state can occur in the absence of rapid bacterial cell growth by transferring, without dilution, stationary-phase bacterial cells grown in LB into LPM pH 5.5 medium. It is anticipated that such a media shift allows for little or no bacterial cell division since overnight growth in LB medium results in a final cell density that is 5-fold greater than that obtained in LPM pH 5.5 medium (5×10^9^ CFU/ml versus 1×10^9^ CFU/ml, respectively). Transfer of hypervirulent strains *S.* Choleraesuis χ3246 and *S.* Bovismorbificans 158 from LB to LPM pH 5.5 medium, without dilution, resulted in a rapid transformation from the less-virulent to hypervirulent state as evidenced by a 500- to 1000- fold increase in i.p. CI value within 4 h post-transfer (Table 3). No measurable increase in CFU (5×10^9^/ml) or optical density (OD_600_) was observed over the 10 h time course in permissive medium (LPM pH 5.5), suggesting little or no bacterial growth is required for the induction of hypervirulence. Conventionally virulent *Salmonella* reference strain 14028 showed no marked increase in virulence after media switch. Taken together, these data indicate that the induction of hypervirulence is rapid and rapidly reversible, and does not require vigorous bacterial cell growth.

**Table 3:** Comparison of virulence states between hyperinfectious and conventionally virulent salmonellae following transfer from nonpermissive to permissive conditions for the hypervirulent phenotype.

		Virulence (i.p. CI)					
		Time post-transfer (h)					
Strain^a^	Serovar	0	1	2	4	8	10
χ3246	*S.* Choleraesuis	0.0003	0.002	0.008	0.148	0.568	0.570
158	*S.* Bovismorbificans	<0.0003	0.015	0.028	0.296	0.592	0.813
14028	*S.* Typhimurium ref. strain	1.14	1.49	1.49	1.68	1.63	1.93

a Hyperinfectious *S.* Choleraesuis χ3246 and *S.* Bovismorbificans 158 as well as conventionally virulent *S.* Typhimurium reference strain 14028 were grown overnight in LB medium. The stationary-phase cells were transferred without dilution, into permissive conditions for the hypervirulent phenotype (LPM pH 5.5 medium). Virulence was assessed as a function of time (h) post-transfer to LPM pH 5.5 medium via i.p. competitive index (CI) virulence assays as in Table 1.

### Environmental conditions that confer a growth advantage to hyperinfectious salmonellae in vivo are associated with a growth disadvantage in vitro

**Figure 3:**
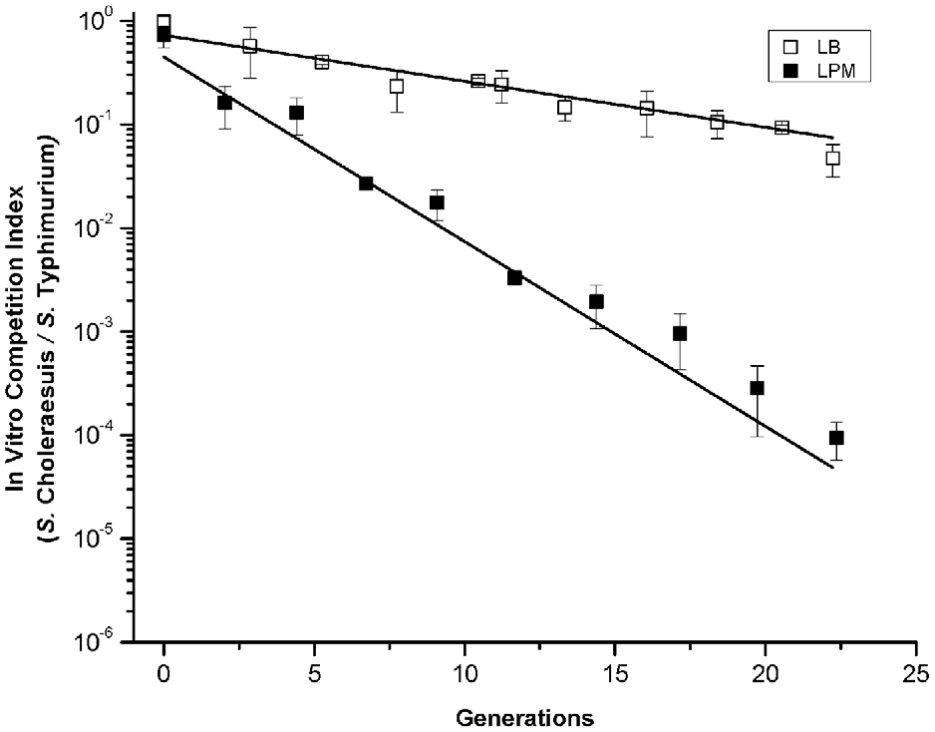
Comparison of growth rates between hyperinfectious and conventionally virulent salmonellae grown under in vitro conditions that are permissive for hypervirulence. An equivalent dose of hyperinfectious *S.* Choleraesuis χ3246 and conventionally virulent *S.* Typhimurium reference strain 14028 (5×10^7^ CFU/ml) were co-cultured in either permissive (LB; open boxes) or nonpermissive (LPM pH 5.5 medium; closed boxes) conditions for the hypervirulent phenotype, following prior growth individually in the same medium. Cell aliquots were sampled for CFU at the cell generation (cell doubling) indicated. Bacterial cells were obtained from, and maintained in, exponential phase cultures diluted periodically such that the cell number was constant at each sampling point. The in vitro competition index is the relative ratio of test strain/reference wild-type strain recovered from the co-culture divided by the input ratio. The values represent the relative ratio of *S.* Choleraesuis/*S.* Typhimurium obtained from 3 independent cultures with the standard error bars designated.

Expression of virulence functions that confer hypervirulence during the infective process may be deleterious to growth outside of the host. Thus, we questioned whether environmental conditions that conferred a growth advantage to hyperinfectious strains in vivo are associated with a growth disadvantage in vitro relative to conventionally virulent *Salmonella*. Hyperinfectious *S.* Choleraesuis χ3246 and conventionally virulent *S.* Typhimurium reference strain 14028 were grown in competition under conditions that were either permissive (LPM pH 5.5 medium) or nonpermissive (LB medium) for hypervirulence. An equivalent dose of both *Salmonella* strains (5×10^7^ CFU/ml) were co-cultured in either LPM pH 5.5 or LB medium following prior growth individually in the same medium. *S.* Choleraesuis χ3246 was outcompeted in the mixed population to a far greater extent in LPM pH 5.5 medium than in LB medium (Figure 3). These data indicate that growth under environmental conditions that fully recapitulate the hypervirulent state obtained after in vivo passage is detrimental to bacterial fitness in vitro- suggesting the possibility that virulence functions favorable for in vivo growth are unfavorable ex vivo.

### The induction of hypervirulence is associated with an increased capacity to provoke macrophage cell death relative to conventionally virulent strains

*Salmonella* infection of macrophages provokes a caspase-mediated proinflammatory cell death program, termed pyroptosis [49], [66], [67]. Here we examined whether hyperinfectious salmonellae are associated with an increased capacity to initiate macrophage cell death versus conventionally virulent strains. Hyperinfectious strains (*S.* Choleraesuis χ3246 and *S.* Bovismorbificans 158) and conventionally virulent *S.* Typhimurium reference strain 14028 were grown under conditions that were permissive (LPM pH 5.5 medium) or nonpermissive (LB medium) for hypervirulence, and used to infect RAW264.7 murine macrophage cell cultures at a multiplicity of infection (MOI) of 10∶1 or 100∶1. A crystal violet dye retention assay was used to assess the degree of *Salmonella* cytocidal activity within cultured macrophages, measured spectrophotometrically 20 h after infection [49], [50]; high cytocidal activity is associated with low dye retention and vice versa. Infection with hyperinfectious strains (*S.* Choleraesuisχ3246and *S.* Bovismorbificans 158) resulted in a dose-dependent increase in cytocidal activity after prior growth in LPM pH 5.5 relative to LB medium (2.8-fold and 1.5-fold, respectively; MOI of 100∶1; Table 4). In contrast, infection with conventionally virulent *S.* Typhimurium reference strain 14028 resulted in a dose-dependent decrease in cytocidal activity after prior growth in LPM pH 5.5 medium relative to LB (2.5-fold; MOI of 100∶1). The differences in cytocidal activity between hyperinfectious strains grown in LB versus LPM pH 5.5 relative to that observed with *S.* Typhimurium 14028 were statistically significant (*P*<0.05). Taken together, these data establish that hyperinfectious strains are associated with an increased capacity to provoke macrophage cell death relative to conventionally virulent strains; the induction of cytocidal activity occurs in a dose-, media-, and strain- dependent fashion.

**Table 4:** Comparison of hyperinfectious and conventionally virulent salmonellae cytocidal activity upon infection of cultured macrophages.

		Cytocidal activity (A_577_)^a^			
		LB		LPM pH 5.5	
		MOI			
Strain	Serovar	10	100	10	100
χ3246	*S.* Choleraesuis	0.561	0.261	0.297*	0.094*
158	*S.* Bovismorbificans	0.246	0.226	0.286	0.151*
14028	*S.* Typhimurium ref. strain	0.328	0.102	0.560	0.251

a Hyperinfectious *S.* Choleraesuis χ3246 and *S.* Bovismorbificans 158 as well as conventionally virulent *S.* Typhimurium reference strain 14028 were derived from stationary phase cultures under permissive (LPM pH 5.5 medium) or nonpermissive (LB medium) conditions for the hypervirulent phenotype. Cultured RAW264.7 murine macrophage cells were infected with bacteria at a multiplicity of infection (MOI) of 10∶1 or 100∶1 At 20 h post-infection, macrophages were stained with crystal violet, and bacterial cytocidal activity was quantified spectrophotometrically (577 nm) as described in *Materials and Methods*; high cytocidal activity is associated with low dye retention. Data given are representative absorbance values derived from each condition performed in triplicate. Standard error of triplicate means is <20%.

*: Designates statistical significance for changes in cytocidal activity of hyperinfectious strains grown in LB versus LPM pH 5.5 medium relative to that found with reference strain *S.* Typhimurium 14028. Cytocidal activity was analyzed using analysis of variance; the change in cytocidal activity of the hyperinfectious *S.* Choleraesuis and *S.* Bovismorbificans were individually contrasted to the change in cytocidal activity of *S.* Typhimurium 14028 at each dose level. A significance level (*P*) of less than 0.05 was considered to be statistically significant.

### Infection of cultured macrophage cells with hyperinfectious strains is associated with an altered host innate immune cytokine response

**Figure 4:**
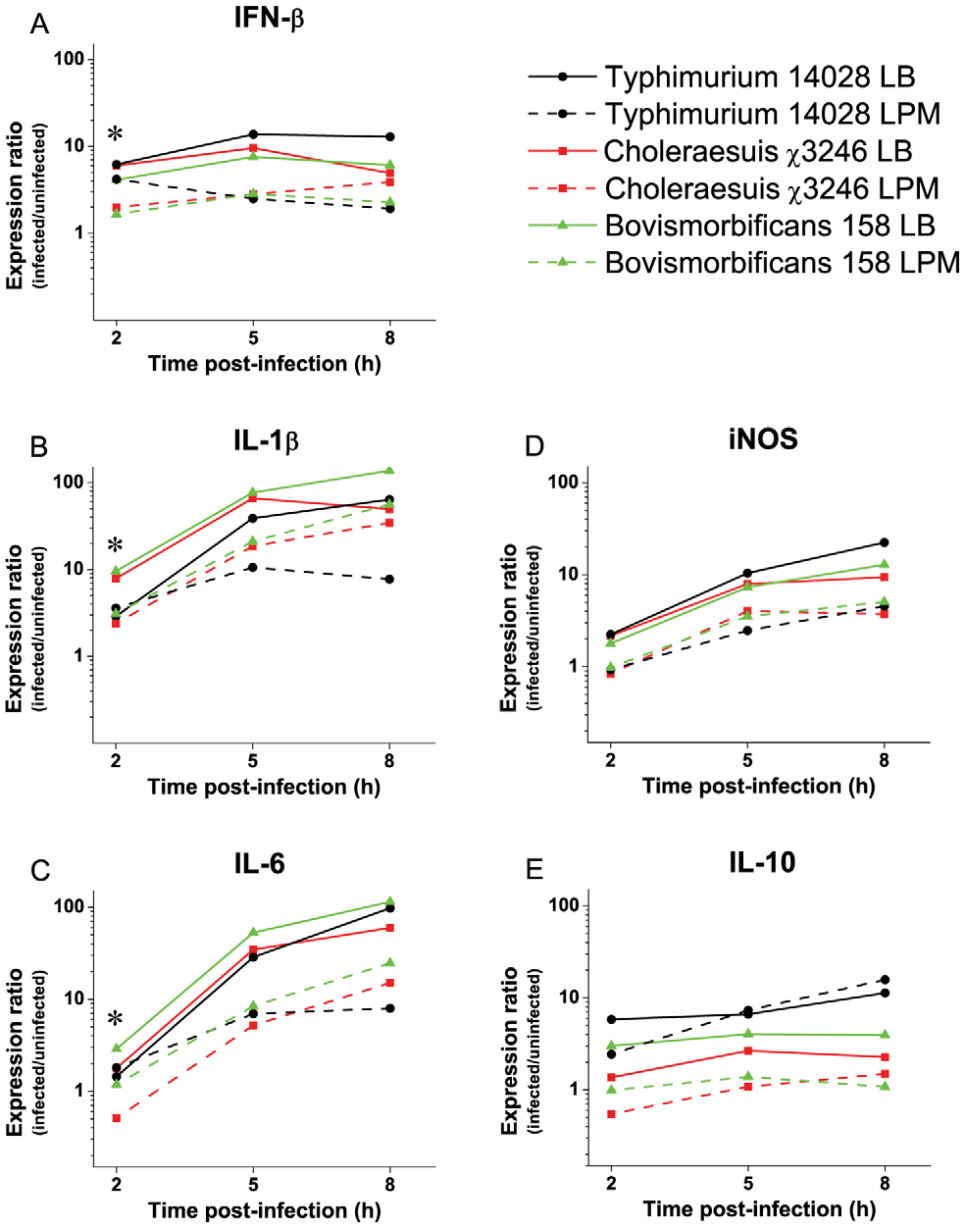
Comparison of cytokine transcript levels in cultured macrophages infected with hyperinfectious and conventionally virulent salmonellae. Innate immune cytokine transcript levels were examined from cultured RAW264.7 murine macrophages infected with hyperinfectious *S.* Choleraesuis χ3246, *S.* Bovismorbificans 158 or conventionally virulent *S.* Typhimurium reference strain 14028 grown under permissive (LPM pH 5.5; dotted lines) or nonpermissive (LB; solid lines) conditions for the hypervirulent phenotype. (A) IFN-β; (B) IL-1β; (C) IL-6; (D) iNOS; (E) IL-10. Bacterial cells derived from stationary phase cultures containing either LB or LPM pH 5.5 medium were used to infect cultured RAW 264.7 murine macrophage cells as described in *Materials and Methods*. The bacteria were centrifuged onto cultured monolayers at 1,000× *g* for 10 min at room temperature, after which they were incubated for 30 min at 37°C in a 5% CO_2_ incubator (*t* = 0 time point). The coculture was washed once and incubated for 45 min with gentamicin (100 µg/ml) at 37°C in a 5% CO_2_ incubator, washed once with pre-warmed cell culture medium, and incubated with gentamicin (10 µg/ml) to the time points indicated (2, 5 and 8 hr). Total RNA was isolated from infected cultured RAW 264.7 murine macrophage cells, and from mock-infected controls as described in *Materials and Methods*. RNA samples were analyzed by reverse transcription and real-time qPCR for: IFN-β; IL-1β; IL-6; iNOS; and IL-10 expression as described in *Materials and Methods*. Relative target gene transcripts were normalized to the level of the GAPDH gene, relative to the average of the normalized values obtained for uninfected RAW 264.7 cells. Values given were obtained from triplicate wells SE <22%. Although reduced stimulation of all cytokine transcripts tested was observed upon infection with both hyperinfectious and conventionally virulent strains grown in LPM pH 5.5 medium relative to that exhibited in LB medium (*P*<0.05), only hyperinfectious strains exhibited a significant reduced stimulation of IFN-β, IL-1β and IL-6 transcript levels at the 2 h infection time point (2.5- to 3.5- fold; *P*<0.05). ^*^Designates statistical significance for those measures that are specific to hypervirulent strains after growth in LPM pH 5.5 medium relative to that exhibited in LB medium (*P*<0.05).

Recognition of conserved pathogen associated molecular patterns (PAMPs) by host-cell pattern recognition receptors (PRRs) activates signaling pathways leading to the stimulation of the innate immune response, characterized by the production of cytokines and interferon system gene products and their potent antimicrobial actions [68]–[72]. To understand the mechanistic basis of hypervirulence, we examined whether infection of cultured RAW264.7 macrophage cells with hyperinfectious strains is associated with an altered innate immune cytokine response. For this analysis, we assessed the relative transcript levels of cytokine and interferon (IFN) system genes known to be induced during *Salmonella* infection including; the type I IFN system gene, IFN-β [50], [53]; the inflammatory and acute phase response genes, interleukin-1 beta (IL-1β) and IL-6 [53], [73]–[76]; inducible nitric oxide synthase (iNOS), a known target of IFN and cytokine signaling required for resistance to *Salmonella* infection [53], [77]–[79]; and IL-10, an inhibitory modulator of the inflammatory response [74], [75], [80], [81]. Hyperinfectious strains (*S.* Choleraesuis χ3246 and *S.* Bovismorbificans 158) and conventionally virulent *S.* Typhimurium reference strain 14028 were grown under conditions that were permissive (LPM pH 5.5 medium) or nonpermissive (LB medium) for hypervirulence, and used to infect cultured RAW264.7 murine macrophage cells. At 2, 5 and 8 h post-infection, RNA was derived from cultured cells and used to assess relative cytokine transcript levels in infected versus uninfected cells. Three salient observations were made (Figure 4): 1) Although reduced induction of all cytokine transcripts tested was observed upon infection with both hyperinfectious and conventionally virulent strains grown in LB versus LPM pH 5.5 medium (*P*<0.05), only hyperinfectious strains exhibited significant reduced stimulation of IFN-β, IL-1β, and IL-6 transcript levels at the 2 h infection time point (2.5- to 3.5- fold; *P*<0.05). 2) The reduced stimulation of IL-1β and IL-6 exhibited by hyperinfectious strains at the 2 h time point was followed by a 14- to 30- fold induction at the 8 h time point. 3) Hyperinfectious strains exhibited significantly reduced stimulation of IL-10 relative to *S.* Typhimurium 14028 irrespective of LB or LPM pH 5.5 growth conditions (*P*<0.05); such reduced stimulation was most pronounced at later infection time points (i.e., 10-fold at *t* = 8 h under LPM pH 5.5 conditions). These data indicate that hyperinfectious strains confer altered kinetics/magnitude of the innate immune cytokine responses that coordinate bacterial clearance via stimulation of signaling receptors and resultant cellular activation and the induction of effector mechanisms; e.g., Toll-like receptor recognition/signaling; inflammasome activation; myeloid cell recruitment; and T cell activation [82]–[84].

### Gene expression analysis of *Salmonella* hyperinfectious strains

**Figure 5:**
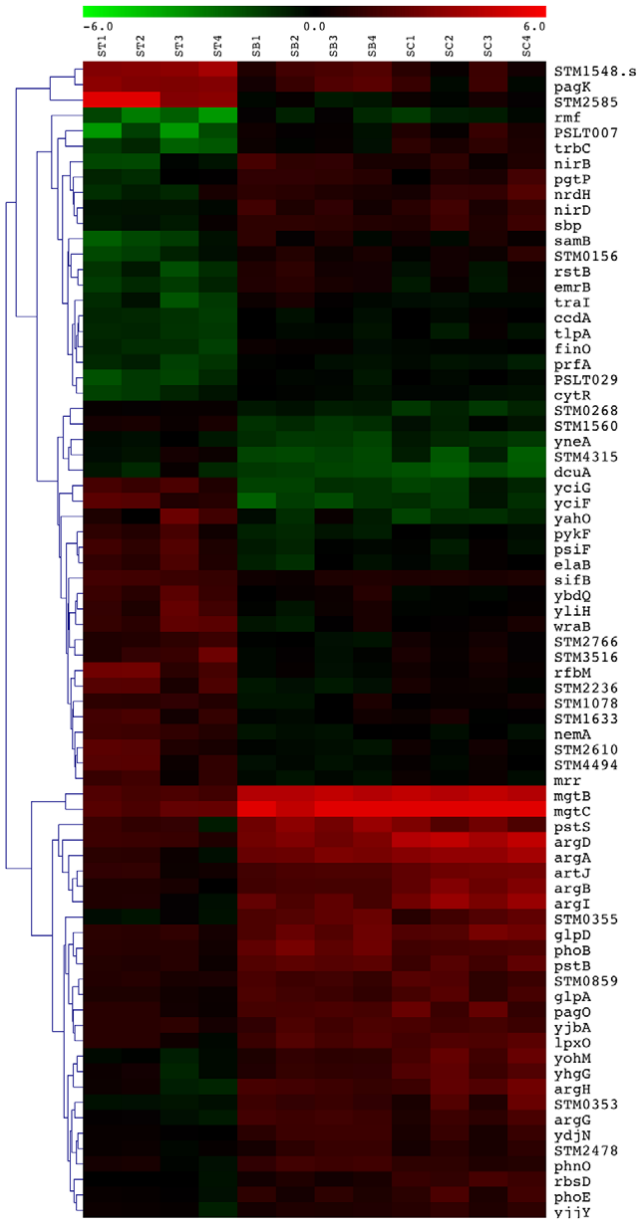
Transcriptome analysis of hyperinfectious strains. Gene expression analysis was performed to identify bacterial gene transcripts that were significantly altered in hyperinfectious strains under LPM pH 5.5 versus LB conditions, and not altered, or altered to the same extent, in a conventionally virulent strain. Hyperinfectious strains (*S.* Bovismorbificans 158 [SB] and *S.* Choleraesuis χ3246 [SC]) and *S.* Typhimurium reference strain 14028 [ST] were grown overnight in LB medium, pelleted and washed in 0.15M NaCl, and split without dilution into two cultures containing either LB or LPM pH 5.5 medium. The cultures were incubated with aeration for 4 h, after which approximately 2.5×10^10^ cells were pelleted via centrifugation. RNA derived from these bacterial cells was used to assess relative transcript levels in bacterial cells via hybridization to a custom *Salmonella* Affymetrix Genechip as described in *Materials and Methods*. Each of the 12 columns of the heat map represents an LPM/LB ratio with four pairwise comparisons provided for each strain. Two criteria were used as a cutoff to identify the genes that were significantly altered in hyperinfectious strains (SB; SC) under LB versus LPM pH.5.5 conditions, and not altered, or altered to the same extent, in a conventionally virulent strain (ST); i.e., at least a 2-fold expression change in SB, SC or ST; and a 0.05 false discovery rate (FDR) when comparing log_2_ LPM/LB ratios values for SB and SC versus ST. Heat maps were generated from the resultant list of genes using The Institute for Genomic Research MultiExperiment Viewer (MeV), version 4.7 [54]. All expression experiments were done in two biological replications.

Gene expression analysis was performed to identify bacterial gene transcripts that were significantly altered in hyperinfectious strains under LPM pH 5.5 versus LB conditions, and not altered, or altered to the same extent, in a conventionally virulent strain. We established that transfer of hypervirulent strains from LB to LPM pH 5.5 medium resulted in a transformation from the less-virulent to hypervirulent state within 4 h post-transfer (Table 3) before proceeding with additional observations. *S.* Choleraesuis χ3246 and *S.* Bovismorbificans 158 were grown overnight in LB medium and transferred, without dilution, to LPM pH 5.5 medium. At 4 h post-transfer, RNA was derived from bacterial cells and used to assess relative transcript levels in cells grown in LPM versus LB via hybridization to a custom *Salmonella* Affymetrix Genechip (see *Materials and Methods*). Microarray analysis revealed that, 4 h post-transfer from LB to LPM pH 5.5 medium, hyperinfectious strains displayed distinct transcriptional responses versus those observed in a conventionally virulent strain (Figure 5; Table S1). At least 3 distinct classes of differentially-regulated genes are represented, including those under the control of the PhoP/PhoQ regulatory system, a global regulator of *Salmonella* virulence [62], [85]–[87]; the PhoR/PhoB regulatory system involved in nutrient (phosphate) stress [88], [89]; and the ArgR regulatory system involved in arginine metabolism including acid stress [90]–[94] (Table 5). Although differential regulation of these genes was observed in both hypervirulent and conventionally virulent strains following transfer from LB to LPM pH 5.5 medium, the degree to which gene expression is altered differs significantly between them. For example, several representative genes show a higher level of induction in hypervirulent strains relative to conventionally virulent strains (*mgtBC;* Mg^2+^ transport [PhoP/Q]; *phoB;* PO_4_^2−^ transport [PhoR/B]; *argA; artJ;* arginine metabolism [ArgR]). Conversely, other PhoP/Q activated genes show a lower level of induction (*pagK*; *sifB;* SPI-2 effectors) or repression (*rtsA*; SPI-I activator) in hypervirulent strains relative to that found in conventional virulent strains. Increased induction of virulence functions involved in cellular physiology and metabolism (*mgtBC; phoB; argA*) in combination with repression of SPI-1 virulence functions involved in invasion after bacterial entry into host cells (repression of the *hilA* activated SPI-1 regulatory cascade via *rtsA* down-regulation; *phoB* up-regulation [reviewed in [95]]) may increase the capacity of hypervirulent strains to undergo in vivo adaptation.

**Table 5:** Bacterial gene transcripts that were specifically altered in hyperinfectious strains under permissive conditions for the hypervirulent phenotype.

Gene number	Gene symbol	Log_2_ LPM/LB ratio a			Description
		ST	SB	SC	
PhoP/PhoQ					
STM4286	*lpxO*	0.54	1.74	1.88	Lipid A modification [139].
STM3763	*mgtB*	1.74	4.34	4.40	Magnesium transporter; required for virulence [140].
STM3764	*mgtC*	2.18	5.13	5.34	Magnesium transport; required for intramacrophage survival and long term systemic infection [141].
STM2585	*pagJ*	4.26	−0.27	0.24	SPI-2 effector; translocated to macrophage cytoplasm [142].
STM1867	*pagK*	3.12	1.47	0.61	SPI-2 effector; translocated to macrophage cytoplasm [142].
STM1862	*pagO*	0.67	1.71	1.89	Homology to *pagO* in *Klebsiella pneumoniae* drug/metabolite exporter [143].
STM0397	*phoB*	0.81	2.50	1.72	Response regulator of PhoR/B regulon; represses *hilA* activated SPI-1 effectors [140], [144].
STM4315	*rtsA*	0.04	−1.62	−1.39	Activates *hilA* and downstream SPI-1 effectors; required for cell invasion [88], [145].
STM1471	*rtsB*	−1.16	0.72	−0.03	Sensory histidine kinase; acts on PhoQ to control PhoP regulated genes [95].
STM1602	*sifB*	1.47	0.56	0.68	SPI-2 effector; translocated to macrophage cytoplasm [146].
STM0366	*yahO*	1.22	−0.43	−1.13	Modification of cell envelope [147].
STM0614	*ybdQ*	1.46	0.37	−0.09	Universal stress protein [144].
PhoB/PhoR					
STM4287	*phnO*	0.16	1.43	0.96	Regulator of phosphocarbonate breakdown [148].
STM0397	*phoB*	0.81	2.50	1.72	Response regulator of PhoR/B regulon; represses *hilA* activated SPI-1 effectors [140], [144].
STM0320	*phoE*	0.03	0.87	1.26	Outer membrane pore protein induced in phosphate limiting conditions [149].
STM0384	*psiF*	1.34	−0.40	−0.23	Phosphate inducible starvation protein [148].
STM3854	*pstB*	0.74	2.12	1.81	High affinity phosphate transporter [148].
STM3857	*pstS*	0.81	3.08	2.38	Induced in macrophage; regulates *hilA* through *phoB* [89].
STM4226	*yjbA*	0.89	1.59	1.60	Induced during macrophage infection [150]; also known as *psiE*.
ArgR					
STM2992	*argA*	0.51	2.71	3.50	N-acetylglutamate synthase [91].
STM4122	*argB*	0.56	1.64	2.74	Acetylglutamate kinase [91].
STM3468	*argD*	1.26	2.84	4.31	Bifunctional N-succinyldiaminopimelate-aminotransferase/acetylornithine transaminase protein [91].
STM3290	*argG*	−0.17	1.54	1.24	Arginosuccinate synthase [91].
STM4123	*argH*	−0.20	1.53	2.10	Arginosuccinate lyase [91].
STM4469	*argI*	0.36	2.05	3.19	Ornithine transcarbamylase [91].
STM0887	*artJ*	0.78	1.76	2.61	Arginine transport system component [91].
Other virulence-associated genes					
STM4077	*yneA*	−0.26	−1.31	−0.96	Involved in quorum sensing; encodes periplasmic receptor for AI-2 [151]; also called *lsrB*.
STM2084	*rfbM*	2.03	−0.13	0.33	Involved in O-antigen synthesis [152]; also known as *manC*.

a The log_2_ LPM/LB gene expression ratios values for conventionally virulent *S.* Typhimurium (ST), and hypervirulent *S.* Bovismorbificans (SB) and *S.* Choleraesuis (SC) strains were determined as described in *Materials and Methods*.

### Infection with hyperinfectious salmonellae leads to increased killing of vaccinated animals

*Salmonella* live attenuated vaccines that contain mutations in the DNA adenine methylase (*dam*) confer cross-protective immunity against virulent challenge with heterologous *Salmonella* strains in murine, avian, and bovine models of salmonellosis [19], [96]–[99]. Here, we assessed whether growth of hyperinfectious strains under permissive conditions for hypervirulence (LPM pH 5.5 medium) leads to increased killing of vaccinated animals. Mice immunized with a *dam* mutant vaccine were more susceptible to infection with hyperinfectious strains grown under LPM pH 5.5 versus LB medium in four of five hyperinfectious *S.* Choleraesuis and *S.* Bovismorbificans strains tested (*P*<0.05) (Table 6). The lone exception is *S.* Choleraesuis χ3246 to which the vaccine conferred poor efficacy under either media tested, although a similar trend was observed (*P* = 0.20). No change in protection was observed in vaccinated animals following challenge with conventionally virulent *S.* Typhimurium reference strain 14028 grown under either media condition. These data indicate that hyperinfectious salmonellae exhibit increased killing of vaccinated animals, suggesting that immunized populations are more susceptible to infection by strains bearing the hypervirulent phenotype.

**Table 6:** Comparison of disease susceptibility in vaccinated mice infected with hyperinfectious and conventionally virulent salmonellae.

		Survivors/Total	
Strain^a^	Serovar	LB	LPM pH 5.5
χ3246	*S.* Choleraesuis	11/41	8/56
3	*S.* Choleraesuis	14/20	3/23*
158	*S.* Bovismorbificans	16/20	10/24*
174	*S.* Bovismorbificans	19/22	10/23*
225	*S.* Bovismorbificans	18/18	7/20*
14028	*S.* Typhimurium ref. strain	20/20	19/22

a BALB/c mice orally immunized with a live, attenuated *dam* mutant *S.* Typhimurium 14028 vaccine [98]. Vaccinated mice challenged with a dose of 100 LD_50_ of hyperinfectious salmonellae derived from stationary phase cultures under conditions that were permissive (LPM pH 5.5 medium) or nonpermissive (LB medium) for the hypervirulent phenotype. Nonvaccinated control mice (25/group) all died by day 21 post-infection. Conventionally virulent *S.* Typhimurium reference strain 14028 was used in all studies for comparison.

*: Designates statistical significance for the number of survivors obtained after *dam* mutant *Salmonella* vaccinated animals were challenged with salmonellae grown in LB medium versus LPM pH 5.5 medium. Statistical significance for difference in proportions was calculated using Chi-square tests; a significance level (*P*) of less than 0.05 was considered to be statistically significant.

## Discussion

Salmonellosis is a principal health concern because of the endemic prevalence of salmonellae in food and water supplies. Recent estimates by the CDC and other sources indicate that *Salmonella* infections cause 1.4 to 1.6 million foodborne illnesses in the U.S. annually at an estimated cost of $2.6 to $14.6 billion [100]–[104]. This health and economic burden will most likely continue to expand due to increased multi-drug resistance and the emergence of new strains that are associated with an increased incidence and/or severity of disease [1], [9], [16]. Insights into the emergence of pathogenic strains have come from animal-passage studies wherein virulence traits are often increased (reversibly) following infection (e.g., hastened colonization, morbidity, and/or mortality; reviewed in [22]–[24]). Here we show that some *Salmonella* strains are considerably more virulent after murine passage relative to other isolates (100-fold decreased LD_50_); and the display of increased virulence traits by bacterial strains after passage does not necessarily equate to hypervirulence. Hyperinfectious strains are among the most virulent salmonellae reported, were restricted to certain serovars, and vaccination conferred poor protection against infection. These strains pose a potential risk to food safety as the parental isolates- from which they were derived- originated from diseased livestock. Molecular characterization of these strains may yield insights into the emergence of hyperinfectious pathogens and the development of intervention strategies for human and animal salmonellosis.

Our findings indicate that salmonellae exhibit intraspecies variation in the development of hyperinfectious strains, as evidenced by the increased likelihood of particular serovars displaying the hypervirulent phenotype than others following murine infection (*S.* Bovismorbificans [11/11] versus *S.* Typhimurium [0/52]). The hypervirulent phenotype was recapitulated in vitro with strains adopting distinct virulence states actuated by prior growth conditions, suggesting that the degree of virulence exhibited by these strains can be modified significantly within different hosts, during different infection states (sub-clinical versus fulminate infection), or after exposure to certain environmental variables. Thus, these strains may lead to disease under some environs but not others [105] (e.g., varied levels of moisture, heat stress, cell density, salts/nutrients). Consequently, in an outbreak scenario, although knowledge of the strain serotype is useful epidemiologically, it may have limited predictive value as to the clinical disease outcome or whether protection will be provided by vaccination.

The mechanistic basis for hypervirulence appears to be the consequence of increased microbial pathogenicity accompanied by microbe-mediated alterations in innate immune cytokine responses in infected animals. This is evidenced by increased microbial cytotoxin (SpvB) production, host tissue site colonization, and cytocidal activity that may coexist in time with a delayed proinflammatory IFN/cytokine response coupled with a diminished proinhibitory (IL-10) cytokine response over the entire infection time course. This immune antagonism strategy is often employed by viruses, interfering with multiple stages of the innate immune response; e.g., disruption of pathogen recognition, downstream signaling pathways, and subsequent repression/inhibition of a number of innate immune responses [69], [106]–[108]. Altered innate immunity during the *Salmonella* infective process can profoundly impact disease outcome as the bacterium must strike a balance between initiating inflammatory responses to promote colonization while avoiding prolonged inflammatory responses that damage host niches occupied by the microbe during infection [109]–[111]. Further, since it is well-established that innate immune responses stimulate the development of adaptive immunity [68], [112], [113], elicitation of an altered IFN/cytokine signature may contribute to the observed increased disease susceptibility in vaccinated animals.

Gene expression analysis revealed that transfer from nonpermissive to permissive conditions for the hypervirulent phenotype (LB versus LPM pH 5.5 medium) resulted in distinct transcriptional responses in hypervirulent strains that were not altered, or altered to the same extent, in a conventionally virulent strain. Three major classes of differentially-regulated genes were identified: those that reside in the PhoP/PhoQ [62], [85]–[87]; PhoR/PhoB [88], [89]; or ArgR regulons [92]–[94] that confer changes in the expression of classical virulence functions (e.g., SPI-1 and SPI-2 effectors) as well as marked changes in cellular physiology and metabolism (nutrient and acid stress response). Such altered regulatory circuitry can contribute in several ways to increased host cell intoxication, immune evasion, and virulence exhibited by hyperinfectious strains. 1) SPI-1 and SPI-2 effectors are known to harbor potent immunomodulatory properties resulting in altered host-cell signaling and resultant innate immune cytokine responses [2], [114]; down-regulation of SPI-1 invasion genes upon bacterial entry (*rstA; phoB*) may optimize survival/proliferation in the *Salmonella* containing vacuole (SCV). 2) Altered physiologic and metabolic changes (*mgtBC; phoB; argA*) are known to impact differences in species-specific lifestyle/behavior; e.g., differential regulation of metabolic, transporter, and motility functions in *Bordetella spp.* is thought to increase the capacity of ex vivo adaptation of *B. bronchiseptica* [115]. Taken together, altered timing, magnitude, and localization of bacterial gene expression can have profound effects on virulence and host immune responses.

Intraspecies variation in the capacity to become hypervirulent may be due to genes encoded by one serotype but not another and/or altered expression of preexisting virulence functions. Acquisition of the *viaB* locus in *S.* Typhi provides genes for Vi capsular biosynthesis (*tviBCDE*) and a regulatory gene (*tviA*) that alters expression of Vi antigen, flagella and the invasion-associated type III secretion system in response to changes in osmolarity [116], [117]. Such altered expression results in reduced inflammatory responses relative to non-typhoidal serotypes, and introduction of the *viaB* locus into *S.* Typhimurium reduces the inflammatory response conferred by this pathogen [118]. Additionally, intraspecies variation in the capacity to become hypervirulent may be due to differential expression (transcriptional re-wiring) of preexisting virulence genes as is the case in cross-species comparisons between BvgA/S regulatory circuit in *B. pertussis* and *B. bronchiseptica* [115] and the PhoP/PhoQ regulatory circuits in multiple Enterobacteriaceae [119], [120]. Thus, intraspecies variation in the capacity to become hypervirulent may be the consequence of gene acquisition and/or altered expression of preexisting virulence functions via alterations in principal regulatory proteins; downstream regulatory proteins; and/or by cis-acting alterations in target genes [121]–[124].

Our findings indicate that the phase-variable phenotypes associated with *Salmonella* hyperinfectious strains are consistent with a phenotypic modulation mechanism as switching between virulence states was rapid and rapidly reversible (non-mutational); did not require vigorous bacterial cell growth; and was responsive to subtle differences in environmental signals resulting in multiple virulence states. Consistent with this suggestion, environmental conditions that stimulate/inhibit the BvgA/BvgS regulatory system in *Bordetella* results in the expression of at least three distinct phenotypic phases that are each associated with a unique gene expression profile thought to play an explicit role in the infectious cycle [125], [126]. This provides a potential means to rapidly adapt to disparate hosts/environments without undergoing irreversible changes in the genome, and may contribute to the maintenance of hyperinfectious strains in nature. Additionally, other serotypes may potentially exhibit hypervirulence in response to passage through certain hosts or exposure to certain environments; and this response may be the case across the microbial realm.

Molecular examination of hyperinfectious strains may provide insights into *i)* differences in disease outcomes between closely-related strains; *ii)* distinct outbreak scenarios that point to the same infectious agent; *iii)* differences in vaccine efficacy between laboratory versus clinical field trials due to the environmental complexity of commercial livestock production systems; and *iv)* the design of vaccines and therapeutic strategies to improve clinical disease outcomes.

### General implications

From a farm-management perspective, it is desirable to understand the management and environmental events that lead to hypervirulence in the context of the production system so that risk management strategies can be implemented to prevent disease. It has been established in livestock that host susceptibility and shedding are dependent on management and environmental events (herd size, adverse weather conditions, equipment failure, labor issues, surface water management) that contribute to compromised host immunity and increased pathogen exposure [7], [12], [13], [127]–[129]. Our studies suggest that livestock production systems have the potential for management and environmental events to alter pathogen virulence. That is, environmental conditions inherent to livestock/feedlots (manure, fecal pack and urine), the influence of diet (high and low protein, fiber, and fat), and/or exposure to sub-therapeutic concentrations of antimicrobials may also inadvertently trigger the induction of salmonellae hypervirulence in livestock.

Epidemiological studies in livestock indicate that the pathogenicity and persistence of *S.* Typhimurium variants range from those that cause infections that are relatively mild and geographically limited, to those that cause small epidemics that circulate in livestock and humans [130], [131], to those that are multi-drug resistant and have the capacity for pandemic spread and increased human and animal disease [132], [133] (reviewed in [134], [135]). Further, although it is common to find salmonellae on farms [7]–[9], a given strain may not be significant from a disease or food safety perspective. Thus, the development of a means to identify strains that are likely to be virulent (or hypervirulent) would provide a better measure of causality and food safety risk and may lead to the identification of targets for immunoprophylaxis.

Such detection may be complicated by the fact that other serotypes may potentially become hypervirulent in response to passage through certain hosts or exposure to certain environments (e.g., cow, pig, manure, surface water); and this response may be prevalent in other pathogens. Thus, molecular characterization of hypervirulence cannot be solely concluded on the basis of culturing in rich media, and more efforts should be given to determining virulence characteristics under more physiological growth conditions and/or in animal models of infection. Of potential benefit to therapeutic efforts are live-animal infection model screens for virulence factors and antibiotics that target microbial functions that confer a growth advantage in vivo relative to that observed in vitro [136]–[138].

Future work will focus on the molecular basis of the emergence of hyperinfectious salmonellae and the development of vaccines, as well as dietary and environmental management strategies to mitigate these potential food-borne contaminants before they cause negative public health impacts and economic losses.

## Supporting Information

Table S1 **List of** ***Salmonella*** **differentially regulated genes in hyperinfectious versus conventionally virulent strains under permissive and nonpermissive conditions for the hypervirulent phenotype.** Gene expression analysis was performed to identify bacterial gene transcripts that were significantly altered in hyperinfectious strains under LPM pH 5.5 versus LB conditions, and not altered, or altered to the same extent, in a conventionally virulent strain as described in Figure 5 legend and *Materials and Methods*.
(XLSX)
